# Neural SDE-based spike control of noisy neurons

**DOI:** 10.1371/journal.pone.0330607

**Published:** 2025-09-16

**Authors:** Fumiya Sato, Masaki Ogura, Airi Sashie, Yang Bai, Masanori Shimono, Naoki Wakamiya

**Affiliations:** 1 School of Engineering Science, Osaka University, Osaka, Japan; 2 Graduate School of Advanced Science and Engineering, Hiroshima University, Hiroshima, Japan; 3 Graduate School of Information Science and Technology, Osaka University, Osaka, Japan; 4 School of Informatics and Data Science, Hiroshima University, Hiroshima, Japan; Lanzhou University of Technology, CHINA

## Abstract

Controlling the spike timing of individual neurons is a fundamental challenge with significant implications for treating neurological disorders. While much research has focused on neural models in low-noise scenarios, real-world applications, such as implantable therapeutic devices, must operate in noisy environments and address the diverse firing patterns of neurons. Leveraging the Izhikevich model, which captures a broad range of firing behaviors, this study proposes a novel method for spike timing control using Neural Stochastic Differential Equations (Neural SDE). The approach iteratively trains external currents to minimize both firing mismatches and timing errors through stochastic gradient descent and back-propagation techniques. Simulations demonstrate that the method achieves precise spike control across various neuron types and noise levels, including regular spiking, bursting, and fast spiking patterns. The approach remains effective even under strong noise perturbations, with particularly high precision observed in early spike events. Unlike conventional methods relying on deterministic dynamics or simplified models, the proposed Neural SDE framework directly accounts for biological noise and complex intrinsic dynamics. This enables the generation of neuron-specific control signals that align spike timings while adapting to individual firing characteristics. These results highlight the method’s generalizability and suggest its suitability for real-world neural control applications, including neuroprosthetics, adaptive stimulation, and closed-loop therapeutic systems.

## Introduction

Neurons constitute the cellular framework of the nervous system, enabling communication via spike-based activity [1]. The precise timing of spikes is critical for neural coding, perception, and motor control [2]. However, neural systems operate under inherently noisy conditions due to synaptic variability, thermal fluctuations, and stochastic ion channel gating [3,4], which must be addressed to ensure reliable control [5–7]. Additionally, neurons exhibit diverse firing patterns, from regular spiking to bursting and chaotic activity, depending on their type and function [8,9], demanding control strategies that are both flexible and robust across dynamic conditions. Disruptions in spike timing are associated with neurological disorders such as epilepsy and Parkinson’s disease. Targeted modulation of spike sequences offers therapeutic potential, particularly in neuroprosthetics and closed-loop stimulation. Robust spike timing control under noisy conditions is therefore essential [10–15].

Controlling neurons with complex firing patterns requires models capable of capturing diverse dynamics [16]. One conventional strategy evaluates the average membrane potential and its variance at spike times [17], focusing on statistical properties rather than precise spike timing. While effective in low-noise settings, this approach becomes unreliable under noisy conditions where fluctuations can significantly affect spike timing [13,18]. Another common approach uses the Leaky Integrate-and-Fire (LIF) model [19], which simplifies neuronal dynamics and performs well for regular spiking. However, its limited flexibility restricts its ability to reproduce the range of firing patterns observed in biological neurons [8,20]. Although spike timing error-based control has achieved low error under noise using the LIF model [21], this model’s simplicity prevents it from replicating more complex behaviors that versatile models like the Izhikevich model can emulate through parameter tuning [22].

The Izhikevich model provides a biologically plausible and computationally efficient framework capable of replicating diverse neuronal firing patterns by tuning its parameters [8,22,23]. This makes it suitable for studying neural dynamics under realistic physiological conditions, where precise spike timing plays a crucial role in communication, synchronization, and plasticity. Disruptions in spike timing are linked to disorders such as Parkinson’s disease and epilepsy, underscoring the importance of precise timing control for neuromodulation and neuroprosthetics. However, the model’s complexity and the presence of intrinsic noise in neural systems present significant challenges for traditional control methods [7].

In this study, we propose a machine learning-based method to control the spike timing of the Izhikevich model under noisy conditions using Neural SDEs [24,25]. Neural SDEs extend Neural Ordinary Differential Equations [26] by incorporating stochastic processes such as Brownian motion, making them well-suited for modeling the intrinsic noise of biological systems and improving control robustness. To enable the application of Neural SDE, we modify the Izhikevich model to ensure non-negative membrane potential derivatives, allowing it to be formulated as a standard stochastic differential equation. This transformation yields a time series of control inputs that induce spiking at desired times [25]. The rationale for adopting Neural SDE lies in its capacity to model continuous-time stochastic dynamics and generate input sequences in noisy environments, both of which are essential for biologically realistic neural control.

The structure of this paper is as follows. Section Methods describes the neuron models, learning methods, cost functions, and simulation parameters used in this study. Section Results and Discussion presents the simulation results and analysis. Finally, Section Conclusion summarizes the main findings and outlines future research directions.

## Methods

In this section, we first introduce the neural cell model considered in this study, elaborating on its dynamics and the modifications made to incorporate noise. Next, we demonstrate how this model is transformed into a standard probabilistic differential equation, facilitating the application of Neural SDE. Furthermore, we propose a detailed methodology for designing input time series using this transformation and the utilization of Neural SDE, outlining the steps involved in the control process.

### Neural cell model

The Izhikevich model [22] comprises two coupled differential equations for scalar variables *v*(*t*) and *u*(*t*), representing the membrane potential and the recovery current of a neuron, respectively:

$$\frac{dv}{dt}=0.04v^{2}+5v+140-u+I,$$ (1)

$$\frac{du}{dt}=a(bv-u).$$ (2)

Here, *v*(*t*) denotes the membrane potential, which reflects the electrical state of the neuron, while *u*(*t*) represents the recovery variable that accounts for the activation of *K*^ + ^ ionic currents, facilitating the return of the membrane potential to its resting state after a spike. The external input current *I*(*t*) is the control variable subject to design in this study, intended to modulate the neuron’s firing behavior.

The parameters *a* and *b* govern the time scale and sensitivity of the recovery variable *u*(*t*) to changes in the membrane potential *v*(*t*), respectively. Specifically, *a* influences the rate at which *u*(*t*) responds to perturbations in *v*(*t*), while *b* determines the degree to which *u*(*t*) is coupled to *v*(*t*). These parameters are crucial for generating a wide variety of firing patterns, including regular spiking, bursting, and chattering.

The membrane potential *v*(*t*) exhibits characteristic behavior within living organisms, rising sharply beyond a certain threshold—a property captured by the quadratic and linear terms in Eq (1). This rapid increase leads to the generation of action potentials or spikes. Eq (2) describes the dynamics of the recovery current *u*(*t*), which acts to restore the membrane potential to its baseline level following a spike by opposing changes in *v*(*t*).

Additionally, the model incorporates a reset mechanism to simulate the refractory period observed in biological neurons. Specifically, when the membrane potential *v*(*t*) reaches a predefined firing threshold $v_{f}$, the following reset dynamics are applied:

$$v(t)\leftarrow c,\;u(t)\leftarrow u(t)+d\;\text{if }v(t)=v_{f}.$$ (3)

Here, *c* is the reset value of the membrane potential, and *d* is the parameter that influences the subsequent behavior of the recovery variable *u*(*t*). This reset mechanism ensures that the neuron does not fire indefinitely and mimics the refractory period necessary for the proper functioning of neural networks. The constants *a*,*b*,*c*,*d* are introduced to generate various firing patterns by altering their values. For instance, different combinations of these parameters can produce regular spiking neurons, bursting neurons, or neurons exhibiting chattering behavior, each of which plays distinct roles in neural computations and information processing.

### Incorporating noise into the model

To better emulate the stochastic nature of biological neural activity, it is essential to incorporate noise into the neural cell model. This incorporation of noise is not only essential for realism but also aligns with previous theoretical analyses which show that the addition of stochastic terms can lead to meaningful bifurcation phenomena and stochastic stability transitions in neuron models [27]. Their work, which focuses on a stochastic variant of the Izhikevich-FitzHugh model, demonstrates how random fluctuations can lead to significant shifts in the system’s dynamics, further supporting the necessity of considering noise in spike control strategies. In this study, we introduce a noise term βdW to the right-hand side of the membrane potential dynamics in Eq (1), where *β* represents the noise amplitude, and *W* denotes a standard Wiener process (Brownian motion). The incorporation of Brownian noise into the membrane potential dynamics not only enables a realistic representation of intrinsic stochastic fluctuations, such as those arising from ion channel gating and synaptic variability, but also allows the model to capture important biological phenomena. For example, spontaneous spiking can emerge in the absence of external stimuli due to the influence of internal noise, reflecting behavior observed in real neurons. Moreover, variability in interspike intervals (ISIs) arises naturally in this stochastic setting, as even repeated trials with identical input can result in distinct spike timings. This trial-to-trial variability aligns with electrophysiological recordings and underscores the biological plausibility of the proposed stochastic modeling framework. This modification leads to the following stochastic differential equation:

$$\frac{dv}{dt}=\max(0,0.04v^{2}+5v+140-u+I)+\beta dW.$$ (4)

This modification ensures that the membrane potential dynamics are subject to random fluctuations, thereby simulating the inherent noise present in biological systems due to various sources such as synaptic noise and ion channel stochasticity. In addition to introducing stochasticity, the use of the max function imposes a non-negativity constraint on the deterministic part of the membrane potential derivative. This constraint ensures that, in the absence of noise, the membrane potential either increases or remains constant, which aligns with the depolarization behavior typically observed during spike initiation. From a modeling perspective, this avoids undesired negative drifts and facilitates the stability and tractability of the learning process under the Neural SDE formulation.

Furthermore, for the purpose of transforming the model into a stochastic differential equation (SDE) suitable for Neural SDE analysis, we impose an additional constraint that the derivative of the membrane potential remains non-negative in the absence of noise. This constraint is implemented using the max function, which ensures that the deterministic part of the membrane potential dynamics does not decrease, thereby maintaining biological plausibility and facilitating the application of stochastic modeling techniques.

The primary objective of control in this study is to design a time series of external input current *I*(*t*) that, within a finite time interval [0,*T*], approximates the desired firing times and firing count as specified. Achieving this objective requires a robust control strategy capable of accounting for the stochastic nature of the neuronal dynamics and the complexity of the firing patterns inherent in the Izhikevich model.

### Transformation to standard stochastic differential equations

The Izhikevich model, as defined by Eqs (1) and (2), involves variable resets and is not a standard stochastic differential equation. To apply Neural SDEs effectively, it is necessary to transform this model into a standard SDE framework. This transformation allows us to leverage the powerful capabilities of Neural SDEs in learning and controlling stochastic processes.

**Proposition 1.** Let us assume β=0. Define the real functions *ϕ* and *ψ* as follows:


$$\phi (x_{1})={\mathrm{mod}}_{v_{f}-c}(x_{1}-c)+c,$$

$$\psi (x_{1},x_{2})=x_{1}+d\frac{x_{2}-\phi (x_{2})}{v_{f}-c},$$

in which ${\text{mod}}_{v_{f}-c}(z)$ represents the minimum *r* that satisfies $z=(v_{f}\;-\;c)q\;+\;r$, where r≥0 and z∈ℤ.

Let the solutions of the modified Izhikevich model (4), (2), and (3) be denoted as *v*(*t*) and *u*(*t*). Additionally, let *n*(*t*) be the number of spikes before time *t*. Furthermore, define the real functions *y*_1_(*t*) and *y*_2_(*t*) as


$$y_{1}(t)=n(t)(v_{f}-c)+v(t),$$

$$y_{2}(t)=u(t)-dn(t).$$

Then, *y*_1_(*t*) and *y*_2_(*t*) are solutions to the differential equations:

$$\frac{dy_{1}}{dt}=\max(0,\phi (y_{1}{)}^{2}+5\phi (y_{1})+140-\phi (y_{1},y_{2})+I),$$ (5)

$$\frac{dy_{2}}{dt}=a(b\phi (y_{1})-\psi (y_{1},y_{2})).$$ (6)

*Proof*: When $v(t)<v_{f}$, we have


$$\phi (y_{1})={\mathrm{mod}}_{v_{f}-c}(y_{1}(t)-c)+c$$

$$={\mathrm{mod}}_{v_{f}-c}(n(t)(v_{f}-c)+v(t)-c)+c.$$

From the definitions and assumptions of the Izhikevich model’s differential Eqs (1), (2), and (3), it follows that $c\leq v(t)\leq v_{f}$, and therefore, $\phi (y_{1}(t))=v(t)-c+c=v(t)$.

Additionally, based on


$$n(t)=\frac{y_{1}(t)-v(t)}{v_{f}-c},$$

we have


$$\psi (y_{1}(t),y_{2}(t))=y_{2}(t)+d\frac{y_{1}(t)-\phi (y_{1}(t))}{v_{f}-c}$$

$$=\left(u(t)-dn(t)\right)+d\frac{y_{1}(t)-v(t)}{v_{f}-c}$$

=u(t).

Furthermore, since dn/dt=0, it follows that *y*_1_(*t*) and *y*_2_(*t*) satisfy the differential Eqs (5) and (6). □

By adding the noise term βdW(t) to the right-hand side of Eq (5), along with Eq (6), we obtain the standard form of a stochastic differential equation. Specifially, this transformation enables the application of Neural SDEs to


$$\frac{dy_{1}}{dt}=\max(0,\phi (y_{1}{)}^{2}+5\phi (y_{1})+140-\phi (y_{1},y_{2})+I(t))+\beta dW(t)$$

for the design of external current *I*(*t*). The transformation provides an analytical foundation for applying Neural SDEs to the spike timing control problem. By converting the original Izhikevich model with reset dynamics into a standard form of stochastic differential equations, we ensure that the resulting dynamics can be handled within the well-established theory of SDEs driven by Brownian motion. This standard SDE form allows for the utilization of Neural SDE frameworks to learn and generate control inputs that can effectively manipulate the firing behavior of the neuron in the presence of noise.

### Learning control input

In this study, the learning is performed using the Neural SDE framework [24], which allows gradient-based optimization in continuous-time models with noise. Since analytically deriving control inputs is difficult due to model complexity and stochasticity, we use stochastic gradient descent to iteratively adjust the external current *I*(*t*). Gradients are computed through backpropagation over stochastic trajectories generated by the Neural SDE solver, enabling the learning process to refine *I*(*t*) to match the desired spike behavior with high temporal precision.

A key to learning in our framework is the design of a loss function that ensures both spike timing precision and appropriate control of the firing count. To achieve this, we introduce three components: spike timing loss $\ell_{1}$, which penalizes misaligned spike times; underfiring penalty $\ell_{2}$, which encourages additional spikes when the actual spike count is too low; and overfiring penalty $\ell_{3}$, which suppresses excessive firings. In our proposed approach, we employ the following three types of loss functions:


$$\ell_{1}(n)=(t_{n}-{\hat{t}}_{n}{)}^{2},$$

$$\ell_{2}=\sum_{t_{N}\leq k\leq t_{\text{size}}}w_{1}(v_{f}-v(hk){)}^{2},$$

$$\ell_{3}=\sum_{t_{\hat{N}}\leq k\leq t_{\text{size}}}w_{2}(v(hk)-c{)}^{2}.$$

Here, *t*_*k*_ represents the actual firing time for the *k*-th event, ${\hat{t}}_{k}$ denotes the desired firing time for the *k*-th event, *h* is the time step when discretizing the model, $t_{\text{size}}$ is the maximum discrete time, *w*_1_ and *w*_2_ are non-negative weights, *N* represents the actual number of firings, and $\hat{N}$ represents the desired number of firings. The discretization of time is performed to provide control inputs of varying magnitudes at each time step, allowing for precise temporal manipulation of the neuron’s firing behavior.

The spike timing loss $\ell_{1}$ is a squared error between actual firing times and desired firing times. This function is expected to drive the learning process towardsu external currents that align the actual firing times with the desired ones, thereby achieving temporal precision in spike control. However, this loss function alone cannot handle cases where the actual number of firings differs from the desired number, leading to uncomputable sets of firing times. In such scenarios, the underfiring penalty $\ell_{2}$ and overfiring penalty $\ell_{3}$ are utilized to address discrepancies in firing counts.

The underfiring penalty $\ell_{2}$ is employed when the firing count is less than desired. It computes the sum of squared errors between the membrane potential *v* and the firing threshold $v_{f}$ after the last actual firing time. The rationale behind this function is to encourage the membrane potential to increase significantly after the last firing time, thereby anticipating and inducing new firing events to meet the desired count. On the other hand, the overfiring penalty $\ell_{3}$ is used when the firing count exceeds the desired count. It computes the sum of squared errors between the membrane potential *v* after the first occurrence of excess firing events and the minimum membrane potential *c*. This function is designed to encourage the learning process to rseduce the firing count back to the desired level if it initially exceeds it, thereby maintaining control over the overall firing behavior. This structure enables not only alignment of actual spike times with desired ones, but also correction of spike count errors, thereby enhancing the temporal precision of the learned input under noise.

In the proposed method, the actual learning loss function, denoted as ℓ, is defined as follows using the three aforementioned loss functions $\ell_{1}$, $\ell_{2}$, and $\ell_{3}$:


$$\ell =\left\{ \begin{array}{cc} \sum_{k=1}^{\hat{N}}\ell_{1}(k), & \text{if }N=\hat{N},\\ \ell_{2}+\sum_{k=1}^{N}\ell_{1}(k), & \text{if }N<\hat{N},\\ \ell_{3}+\sum_{k=1}^{\hat{N}}\ell_{1}(k), & \text{otherwise.} \end{array}\right.$$

This composite loss function is expected to facilitate learning that simultaneously minimizes discrepancies in both firing times and firing counts, thereby achieving precise and reliable spike control.

Stochastic gradient descent is employed to optimize the control input *I*(*t*) based on the specified loss functions. The gradients required for this optimization are calculated using the Neural SDE framework, which efficiently handles the stochastic nature of the neuronal dynamics and the continuous-time modeling of the system. By iteratively adjusting *I*(*t*) to minimize the loss function ℓ, the model learns to generate control inputs that drive the neuron’s firing behavior towards the desired targets, even in the presence of significant noise.

### Objective and simulation settings

We outline the objectives of the simulations conducted in our study and provide specific numerical values for the parameters used in these simulations. The simulations are designed to validate the efficacy of the proposed Neural SDE-based control methodology in achieving precise spike timing control under noisy conditions. The corresponding code for the simulations can be found in [28].

The primary objective of our simulations is to induce the firing of a single neuron at specified desired times using appropriately designed control inputs. This is achieved by periodically inputting external currents *I*(*t*) to the neuron and updating their magnitudes based on gradient calculations derived from the defined loss functions. The goal is to demonstrate that the proposed method can effectively manipulate the neuron’s firing behavior to match the desired spike timings, even in the presence of substantial noise.

Table 1 presents the numerical values of the parameters and the solver used in this report. These parameters were selected to create a controlled simulation environment that closely mimics the conditions under which the proposed methodology would be applied in practical scenarios.

**Table 1:** Simulation parameters and settings.

Parameter	Value
Learning Epochs	100
Batch Size	5
Total Simulation Time	5.0 ms
Desired Firing Times	1.5 ms, 3.0 ms
Discretization Step	0.1 ms
Minimum Control Input	–100 mV
Maximum Control Input	100 mV
Neural SDE Solver	torchsde
Programming Language	Python 3.8.10

In this report, simulations were conducted with four different firing patterns by varying the parameters *a*,*b*,*c*,*d* within the Izhikevich model. These variations allow us to assess the robustness and versatility of the proposed control method across different neuronal behaviors. Table 2 presents the values of *a*,*b*,*c*,*d* used in this report along with the corresponding names for the firing patterns.

**Table 2:** *a*,*b*,*c*,*d* parameters and corresponding firing patterns.

Firing Pattern	*a*	*b*	*c*	*d*
Regular Spiking (RS)	0.02	0.2	–65	8.0
Intrinsically Bursting (IB)	0.02	0.2	–55	4.0
Chattering (CH)	0.02	0.2	–50	2.0
Fast Spiking (FS)	0.1	0.2	–65	2.0

Each firing pattern corresponds to a distinct neuronal behavior, enabling the assessment of the control method’s effectiveness across a spectrum of dynamic responses. For instance, Regular Spiking (RS) neurons exhibit consistent, single spikes in response to sustained input, while Intrinsically Bursting (IB) neurons fire bursts of spikes. Chattering (CH) neurons are characterized by rapid, repetitive firing at high frequencies, and Fast Spiking (FS) neurons fire at very high frequencies with short refractory periods. By evaluating the control method across these diverse patterns, we aim to demonstrate its generalizability and robustness in managing complex neuronal dynamics under noisy conditions.

## Results and discussion

In this section, we present the results of our simulations, illustrating the effectiveness of the proposed Neural SDE-based control methodology in managing spike timing across different neuron types within the Izhikevich model.

### Periodic firing sequence

**Fig 1:**
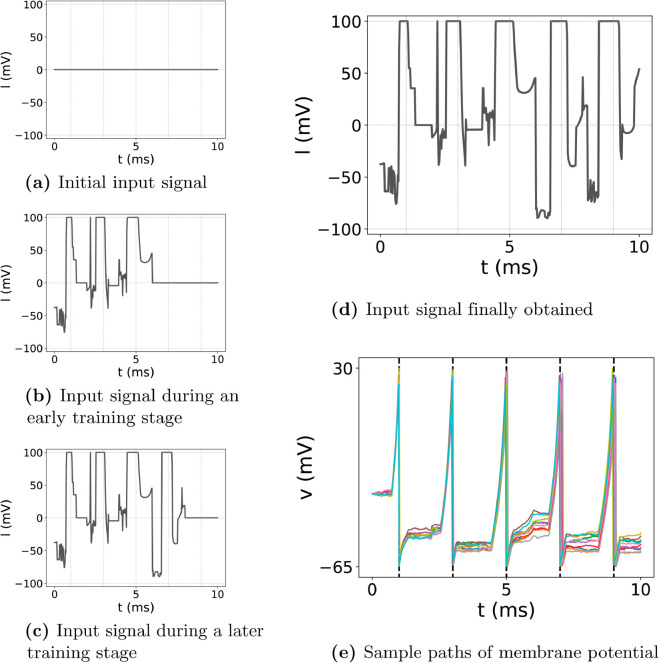
Overview of the process of training the input signal for the case of RS-type neuron. (a)–(c): input signal *u* before, in the early stage of, and the later stage of training. (d): input signal finally obtained. (e): ten sample paths of membrane potential *v* from the RS-type neuron with the obtained input signal.

We first set the desired firing times to ${\hat{t}}_{1}=1$, ${\hat{t}}_{2}=3$, ${\hat{t}}_{3}=5$, ${\hat{t}}_{4}=7$, and ${\hat{t}}_{5}=9$ and then perform our simulations. Fig 1 provides a comprehensive overview of the training process for the input signal tailored to the Regular Spiking (RS) neuron type. Initially (Fig 1a), the input signal is entirely zero from *t* = 0 to *t* = 10, serving as a baseline with no pre-existing bias. As training advances (Fig 1b, 1c), distinct peaks emerge at the intended firing moments, reflecting the neuron’s growing adaptation to the control objectives. By the final stage (Fig 1d), the input signal exhibits five well-defined peaks precisely aligned with all five desired firing times. This progression demonstrates the learning algorithm’s capability to iteratively refine the input, guiding the neuron to achieve the specified spike sequence.

The corresponding membrane potential (Fig 1e) shows the RS neuron firing spikes in close proximity to each targeted time. This alignment underscores the resilience of the control method in handling the stochastic nature of the noisy Izhikevich model. Achieving such precise control under conditions that include noise and inherent model complexity confirms the Neural SDE-based approach as a promising tool for reliable spike timing manipulation. Such precision could prove invaluable in scenarios where subtle temporal patterns are essential, such as restoring normal firing rhythms in neurological disorders.

**Fig 2:**
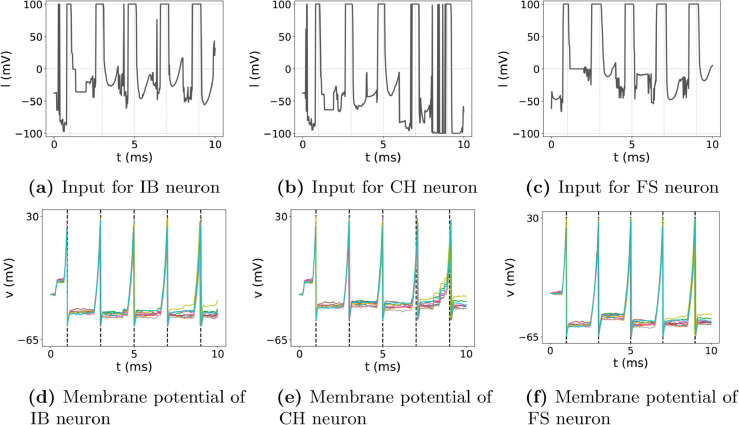
Spking control of IB, CH, and FS neurons. (a)–(c): trained input signals for IB, CH, and FS neurons, respectively. (d)–(f): resulting membrane potentials of the IB, CH, and FS neurons, respectively.

To assess the generality of our approach, we applied the control method to various neuron types: Intrinsic Bursting (IB), Chattering (CH), and Fast Spiking (FS) neurons, as shown in Fig 2. For each neuron type, ten simulations were conducted. Across these simulations, minimal differences were observed among the different neuron types. IB neurons (Fig 2a, 2d) demonstrated targeted firing peaks that closely matched the clarity seen in RS neurons, indicating that the control method effectively manages noise without compromising spike timing. Similarly, CH neurons (Fig 2b, 2e) and FS neurons (Fig 2c, 2f) exhibited input signals and membrane potentials nearly indistinguishable from RS neurons. This consistency across IB, CH, and FS neuron responses demonstrates that our approach can efficiently manage diverse firing patterns and maintain reliability despite neuronal variability. Although the membrane potential trajectories in Fig 2 appear similar across neuron types, this is an intentional outcome of our control objective: enforcing the same set of predefined spike timings. The control inputs shown in Fig 2(a) and 2(c) are individually learned for each neuron type, and the underlying Izhikevich parameters differ as shown in Table 2. This result highlights the method’s ability to generate customized control inputs that compensate for intrinsic differences in neuronal dynamics while producing aligned spike outputs. Therefore, these results collectively indicate that the proposed control method consistently achieves precise spike timings across multiple neuron types, underscoring the broad applicability of the control strategy and suggesting its potential utility in various research and clinical contexts, such as guiding abnormal spike patterns towards normal functional states in neurological conditions.

**Fig 3:**
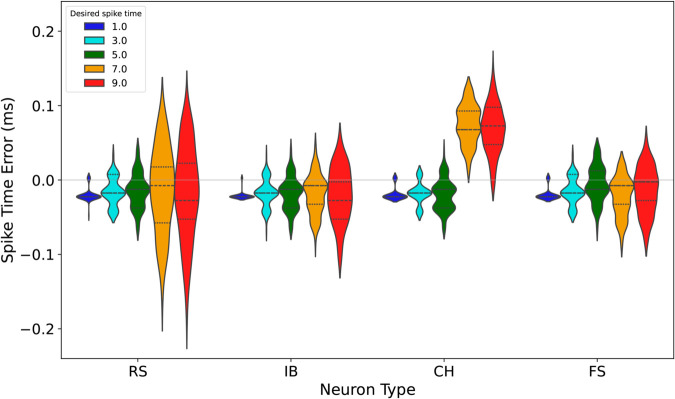
Distribution of firing time errors for RS, IB, CH, FS neuron types, respectively.

To evaluate the control method’s consistency across neuron types, we analyzed the firing time errors from 100 independent simulations for each desired spike separately for RS, IB, CH, and FS neurons (Fig 3). In each firing pattern, five consecutive spikes are controlled, and timing distributions of all five spikes are displayed across 100 trials. Each trial uses a different noise realization to reflect stochastic variability. The *x*-axis represents time, and vertical lines mark the desired spike timings. The results suggest the effectiveness of the proposed method. For the first spike, the mean errors are –0.020 ms, –0.021 ms, –0.019 ms, and –0.020 ms for RS, IB, CH, and FS neurons, respectively, with standard deviations of 0.008 ms, 0.009 ms, 0.010 ms, and 0.008 ms. Medians for all neuron types remain close to –0.023 ms, indicating consistent alignment of the first firing event across neuron types. Similarly, for the second spike, mean errors slightly decrease to –0.016 ms, –0.018 ms, –0.019 ms, and –0.019 ms for RS, IB, CH, and FS neurons, respectively, with standard deviations of 0.007 ms, 0.007 ms, 0.008 ms, and 0.007 ms.

For CH neurons, however, a noticeable increase in mean errors is observed for the 4th and 5th firing events, with values of 0.074 ms and 0.068 ms, respectively. These errors are markedly larger compared to earlier spikes, where mean errors remain close to zero. This trend is likely attributed to the characteristic high-frequency bursting of CH neurons, which compounds variability in the later spikes. As the control method aligns input signals with desired spike times, this inherent dynamic may introduce additional delays or shifts in spike timing during prolonged bursts. Despite this increase, the standard deviations for these events remain moderate, indicating that the method maintains a degree of reliability even under these challenging conditions.

Overall, the results show that the control method achieves precise alignment in early spikes across all neuron types, with increased variability in later spikes. This trend likely results from cumulative uncertainty over time, which is a well-known feature of stochastic systems. The relatively small standard deviations in the first few spikes reflect the method’s effectiveness during initial alignment, while the increased variability in later spikes underscores the intrinsic difficulty of sustaining high-precision control in noisy, biologically realistic settings. Future extensions of the method, such as incorporating multi-step feedback or uncertainty-aware strategies, may help mitigate this variability. Although direct comparisons with existing methods are currently challenging due to differences in models and objectives, the observed control accuracy under noise suggests that the proposed approach offers meaningful effectiveness. Future work will focus on establishing benchmarks to enable more systematic comparisons.

### Random firing sequence

While the performance of the proposed control method has been validated for periodic sequences of desired firing times in previous sections, real-world applications often involve firing sequences that are not periodic but instead irregular or random. To evaluate the method’s effectiveness in such scenarios, we tested it on a random firing sequence with desired firing times set as ${\hat{t}}_{1}=1.6$, ${\hat{t}}_{2}=2.0$, ${\hat{t}}_{3}=3.5$, ${\hat{t}}_{4}=4.2$, and ${\hat{t}}_{5}=9.0$. These timings introduce additional complexity, especially for the second and fourth spikes, which occur less than one millisecond after the preceding spikes, making precise alignment particularly challenging.

**Fig 4:**
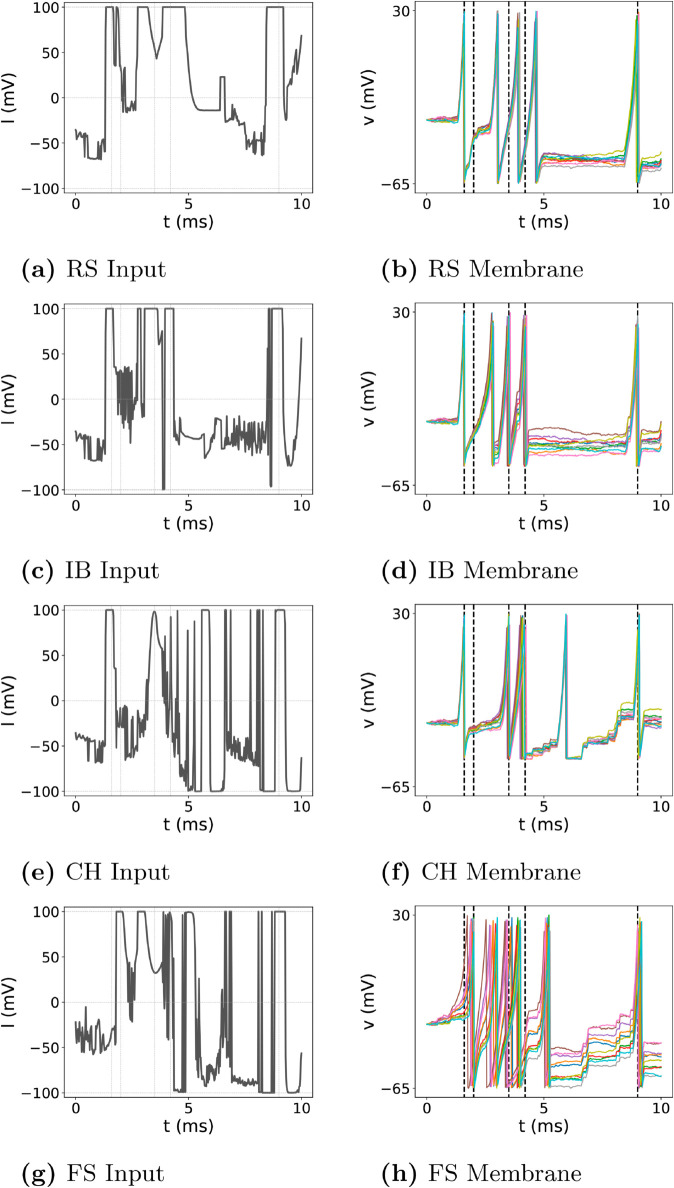
Membrane potentials of four neuron types (RS, IB, CH, FS) in the case of random firing sequences. Each row corresponds to one neuron type: left column for the trained input signal, right column for the resulting membrane potential.

**Fig 5:**
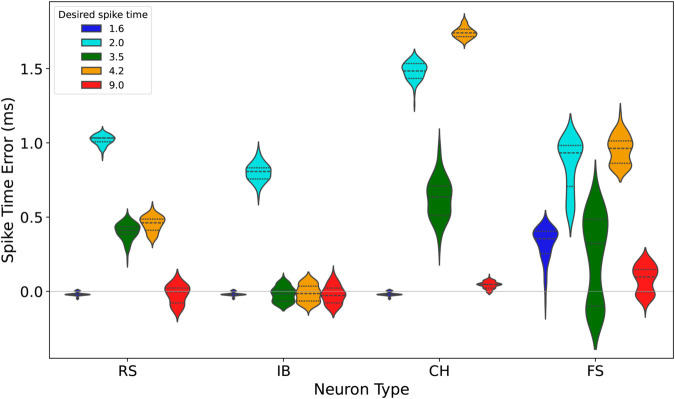
Distribution of firing time errors for RS, IB, CH, FS neuron types, respectively.

The input signals and resulting firing times for each of the RS, IB, CH, and FS neurons are shown in Fig 4. Fig 5 summarizes the firing time errors for each neuron type and spike, using grouped box plots for clarity. For the first spike (*t*_1_), RS, IB, and CH neurons exhibit mean errors of approximately –0.018 ms, with medians around –0.021 ms and narrow standard deviations close to 0.012 ms. This indicates that the control method achieves consistent and reliable performance for the initial spike across these neuron types. However, FS neurons display a significantly larger mean error of 0.321 ms with a standard deviation of 0.113 ms, suggesting greater variability in precision for the first spike in FS neurons. This discrepancy could be attributed to the faster dynamics inherent to FS neurons, which require tighter input control to achieve precise alignment.

The second spike (*t*_2_), occurring just 0.4 ms after the first, proves challenging for all neuron types. RS neurons show a mean error of 1.019 ms with a narrow standard deviation of 0.034 ms. This pattern is consistent across other neuron types, where the errors remain within a similar range. Despite this difficulty, the control method demonstrates a recovery in performance for the third spike (*t*_3_). FS neurons, in particular, show reduced errors compared to earlier spikes, with a mean error of 0.275 ms and a standard deviation of 0.045 ms. CH neurons, while more variable, also display improvement for *t*_3_, with standard deviations decreasing relative to *t*_2_. This indicates the method’s ability to stabilize performance after handling tightly spaced firing events.

The fourth spike (*t*_4_) presents similar challenges to *t*_2_, as it occurs less than one millisecond after the third spike. Here, FS neurons exhibit the largest mean error at 0.315 ms, while RS neurons show slightly better performance, with mean errors around 0.250 ms. The variability for CH neurons increases slightly, with a standard deviation of 0.100 ms. These results suggest that the difficulty in controlling closely spaced spikes is not unique to one neuron type but reflects a general limitation of the current control approach when managing tightly coupled firing events.

For the fifth spike (*t*_5_), the method excels across all neuron types. RS neurons achieve mean errors close to zero, with standard deviations below 0.010 ms, indicating near-perfect alignment with the desired timing. Similar trends are observed for IB and CH neurons, with both achieving consistent precision for the final spike. This success demonstrates the robustness of the control method in handling later spikes within a sequence, even following challenges in earlier timing alignments. The results for *t*_5_ underscore the potential of the method to reliably manage long-range firing sequences, which is critical for applications requiring sustained timing accuracy.

The observed trends highlight both strengths and areas for improvement in the proposed method. The challenges observed for *t*_2_ and *t*_4_ reflect the difficulty in managing closely spaced spikes, particularly under random sequences. This limitation could be addressed by incorporating adaptive learning mechanisms or refining input signal design to better handle these scenarios. On the other hand, the method’s ability to recover performance for *t*_3_ and achieve exceptional precision for *t*_5_ illustrates its overall robustness and adaptability. These findings suggest that the control method is well-suited for managing random firing sequences, albeit with potential for further enhancements.

In summary, while the control method demonstrates strong adaptability to random firing sequences, its performance is not uniform across all spikes. The challenges associated with *t*_2_ and *t*_4_ provide valuable insight into potential refinements, whereas the success at *t*_5_ highlights the method’s potential for long-term precision. Future improvements aimed at enhancing the handling of tightly spaced spikes could further solidify the method’s applicability to complex and irregular neural dynamics.

### Noise impact on spike timing

**Fig 6:**
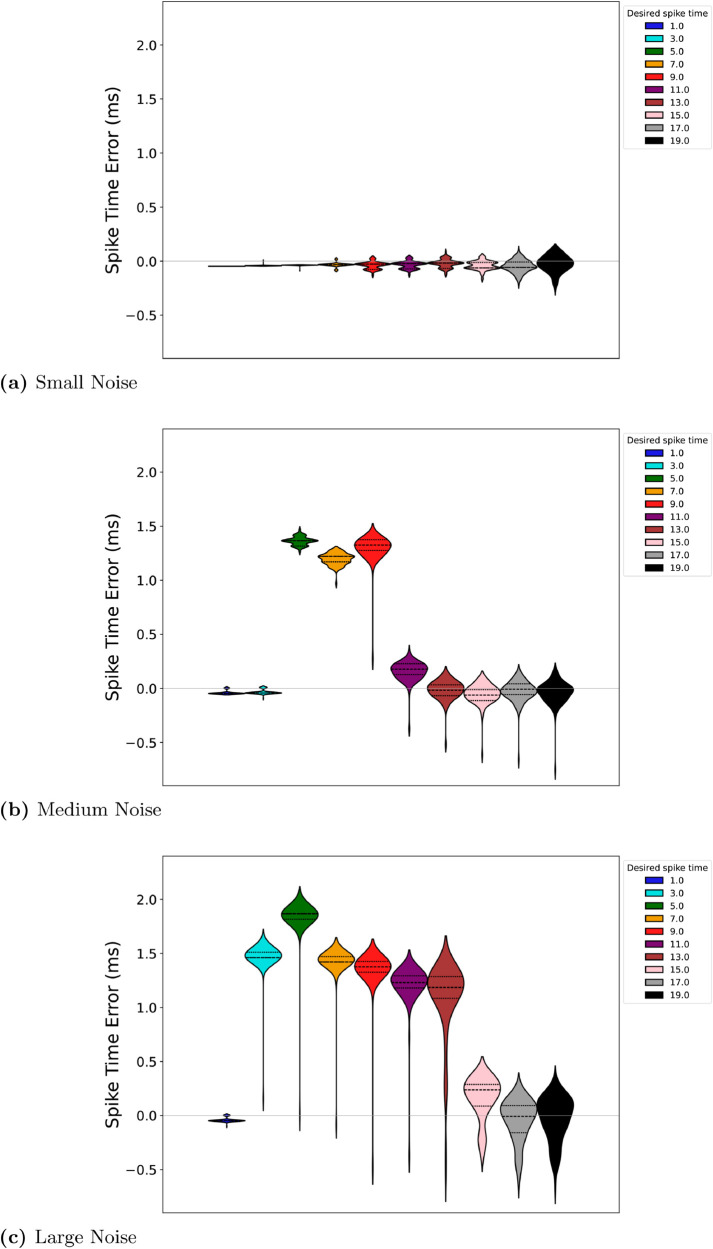
Distribution of firing time errors for RS neurons under different noise levels. Top: small noise, middle: medium noise, bottom: large noise.

To assess the robustness of the proposed control method, simulations were conducted across three noise levels: small (β=0.5), medium (β=1.0), and large (β=2.0). By analyzing the firing time errors for all ten desired spike timings, ${\hat{t}}_{1}=1$, ${\hat{t}}_{2}=3$, , ${\hat{t}}_{10}=19$, this evaluation (see Fig 6) highlights the method’s strengths and identifies areas for improvement under different noise conditions.

When subject to small noise, the method demonstrated high precision across all spike timings, indicating its efficacy in controlled or low-noise environments. Early timings such as ${\hat{t}}_{1}$, ${\hat{t}}_{2}$, and ${\hat{t}}_{3}$ showed mean errors of –0.048 ms, –0.043 ms, and –0.038 ms, respectively, with negligible variability. This level of precision reflects the method’s robustness for fine-grained spike alignment at the onset of the sequence. As the sequence progressed, errors for mid-range timings (${\hat{t}}_{5}$, ${\hat{t}}_{6}$) remained low, at approximately –0.073 ms and –0.068 ms, respectively, despite slight increases in variability. Even for later timings, such as ${\hat{t}}_{9}$ and ${\hat{t}}_{10}$, mean errors were modest, at –0.008 ms and –0.003 ms. These results underscore the method’s reliability and accuracy in environments with minimal noise, making it well-suited for applications requiring precise control, such as in silico or controlled in vitro experiments.

Under moderate noise, the performance of the method showed some degradation, particularly for mid-sequence timings, while retaining reasonable accuracy for early and late timings. Early spikes, such as ${\hat{t}}_{1}$ and ${\hat{t}}_{2}$, maintained relatively small errors at –0.048 ms and –0.043 ms, demonstrating resilience to moderate noise at the onset. However, errors for ${\hat{t}}_{3}$ increased significantly to approximately 1.32 ms, highlighting challenges in maintaining alignment as noise propagated through the sequence. Mid-sequence timings (${\hat{t}}_{5}$, ${\hat{t}}_{6}$) exhibited errors of around 1.18 ms and 1.39 ms, reflecting the sensitivity of these timings to cumulative disturbances. Interestingly, errors for late spikes (${\hat{t}}_{9}$ and ${\hat{t}}_{10}$) stabilized, with mean values around –0.15 ms. These results suggest that while mid-sequence timings require further optimization, the method retains robustness for early and late spikes under moderate noise, making it suitable for applications with tolerable variability.

Under large noise, the method faced substantial challenges, but certain patterns of resilience were observed. For early spikes such as ${\hat{t}}_{1}$, the mean error remained at –0.048 ms, demonstrating robust alignment at the onset. However, errors for subsequent spikes, such as ${\hat{t}}_{2}$ and ${\hat{t}}_{3}$, increased dramatically to 1.46 ms and 1.82 ms, respectively. Mid-sequence timings, such as ${\hat{t}}_{5}$ and ${\hat{t}}_{6}$, experienced significant deviations, with mean errors reaching 1.28 ms and 1.39 ms, reflecting the difficulty of maintaining alignment amidst substantial noise disturbances. Later timings, ${\hat{t}}_{9}$ and ${\hat{t}}_{10}$, showed partial recovery, with mean errors reduced to approximately 0.15 ms. These findings highlight the method’s limitations under high noise conditions but also suggest avenues for improvement, such as adaptive recalibration mechanisms to mitigate noise-induced deviations.

Across all noise levels, a consistent pattern emerged: the method excelled in aligning early spikes with high precision, while mid-sequence timings were most affected by noise. Also, the partial recovery observed in late timings suggests potential resilience mechanisms inherent to the control strategy, which could be enhanced to address cumulative noise effects. While the performance under large noise indicates areas for refinement, the method’s robustness under small and moderate noise conditions demonstrates its practical value for scenarios requiring precise spike alignment.

By incorporating feedback loops, noise-aware adjustments, or dynamic recalibration of input signals, the method could achieve greater stability across all noise levels. These enhancements would extend its applicability to more challenging environments while preserving its strengths in controlled settings. Overall, this evaluation underscores the promise of the proposed method as a versatile tool for neural spike control, particularly in low-to-moderate noise environments, while providing a roadmap for future improvements.

## Conclusion

This study introduced a novel method for controlling spike timing in noisy neural systems using Neural SDEs. By leveraging the Izhikevich model, which replicates diverse firing patterns, our approach achieves robust and precise spike control under varying noise conditions.

Simulation results under small, medium, and large noise intensities revealed that the method maintains high precision for early and late spikes, while mid-sequence spikes remain more sensitive to noise. Across neuron types, the method demonstrated generalizable performance. Although high-frequency bursting patterns such as those in chattering neurons posed greater challenges, control precision remained within tens of microseconds for most conditions.

Unlike traditional deterministic control approaches, our method employs a Neural SDE framework, which naturally captures the intrinsic stochasticity of biological systems. This enables reliable spike timing control even under strong noise, as demonstrated in Fig 6. Furthermore, Figs 3 and 5 confirm the method’s robustness across periodic and non-periodic targets, and across four representative neuron types. Compared to simplified models, our method offers clear advantages in both accuracy and generalizability. These strengths make the proposed approach particularly suitable for real-world applications such as neuroprosthetics, deep brain stimulation, and closed-loop neuromodulation, where spike timing should be controlled reliably in the presence of biological noise.

While this study does not consider coherence or stochastic resonance [29–31], we acknowledge that such phenomena may arise in noisy systems. Future work may incorporate metrics such as CV, SNR, or average energy to investigate their potential impact on control performance. Moreover, theoretical questions regarding the stability and potential chaotic dynamics of the noise-driven Izhikevich model remain open. Extending the framework to hybrid stochastic models with discrete synaptic events or Poisson noise could further improve biological realism and control robustness. Future directions also include adaptive recalibration mechanisms, scaling to larger neural networks, and experimental validation to fully establish the method’s utility as a reliable tool for neural control in both research and clinical settings.
